# Current Progress of Ferroptosis Study in Hepatocellular Carcinoma

**DOI:** 10.7150/ijbs.96014

**Published:** 2024-07-01

**Authors:** Xinyue Zhu, Xudong Sha, Yan Zang, Qiaohui Ren, Shubing Zhang, Dongyue Ma, Lianzi Wang, Junxiao Yao, Xinyi Zhou, Li Yu, Tao Li

**Affiliations:** 1 Department of Clinical Laboratory, the First Affiliated Hospital of Anhui Medical University, Shushan District, No. 218 Jixi Road, Hefei, 230032, Anhui, China.; 2 Department of Pharmacology and Chemical Biology, Shanghai Jiao Tong University School of Medicine, Shanghai, 200025, China.; 3 Anhui Province Key Laboratory of Zoonoses, Anhui Medical University, Hefei, 230032, Anhui, China.

**Keywords:** ferroptosis, hepatocellular carcinoma, molecular mechanism, combined treatment

## Abstract

Ferroptosis, an emerging type of programmed cell death, is initiated by iron-dependent and excessive ROS-mediated lipid peroxidation, which eventually leads to plasma membrane rupture and cell death. Many canonical signalling pathways and biological processes are involved in ferroptosis. Furthermore, cancer cells are more susceptible to ferroptosis due to the high load of ROS and unique metabolic characteristics, including iron requirements. Recent investigations have revealed that ferroptosis plays a crucial role in the progression of tumours, especially HCC. Specifically, the induction of ferroptosis can not only inhibit the growth of hepatoma cells, thereby reversing tumorigenesis, but also improves the efficacy of immunotherapy and enhances the antitumour immune response. Therefore, triggering ferroptosis has become a new therapeutic strategy for cancer therapy. In this review, we summarize the characteristics of ferroptosis based on its underlying mechanism and role in HCC and provide possible therapeutic applications.

## Introduction

Hepatocellular carcinoma (HCC), one of the most prevalent malignancies and a leading cause of cancer-related mortality worldwide 1,2, is characterized by rapid disease onset and progression. HCC accounts for 80%~90% of primary liver cancers 3. HCC is a highly lethal disease due to its poor prognosis, high recurrence rate, rapid progression and therapeutic resistance, although significant advances have been achieved in diagnosis and treatment. Compared with that of noncancer cells, the metabolism of iron in hepatoma cells usually changes due to iron requirements. Accordingly, ferroptosis, a term coined by Dixon et al. in 2012, is an iron-dependent form of cell death 4.

Ferroptosis, a novel type of regulated cell death (RCD), is caused by iron dependence and the lethal accumulation of lipid peroxidation products, which culminates in membrane destruction and cell death 5. Ferroptosis differs markedly from apoptosis and other types of regulated cell death and has distinct morphological and mechanistical features 6. Considerable effort has been devoted to examining the physiological and pathophysiological effects of ferroptosis, which has dramatically expanded our knowledge.

Strikingly, the crux of cancer therapy is how to effectively eliminate cancer cells without impairing normal cells. Inducing cancer cell death, including ferroptosis, is currently the primary treatment in certain contexts 7. Ferroptosis, a unique cell death mechanism, has sparked great attention in cancer research because targeting ferroptosis might provide a novel approach for treating cancers refractory to conventional therapies, such as those with chemotherapy resistance. Therapy resistance occurs due to the presence of some antagonistic mechanisms in cancer cells. Therefore, targeting ferroptosis and attenuating defence systems could provide an intriguing approach for killing therapy-resistant cancers. Iron overload has been observed in HCC, and research based on an increase in susceptibility to ferroptosis in HCC has been well underway.

In this review, we focus on studies of ferroptosis in the setting of HCC. We first summarize the current understanding of the mechanisms of ferroptosis, including its prerequisites and defensive systems. Next, we highlight several mechanisms involved in and underlying ferroptosis in HCC, including those involving noncoding RNA, NRF2, HIF-1α, P53 and metabolic dysregulation. Thirdly, we gather research findings based on an increase in susceptibility to ferroptosis combined with chemotherapy or immunotherapy to treat HCC. Finally, we highlight the challenges and future directions regarding investigations of ferroptosis.

## Ferroptosis: A bird's-eye view

Ferroptosis, which was first proposed in 2012, refers to a form of regulated cell death triggered by excess iron-dependent peroxidation of PUFA-containing phospholipids (PUFA-PLs) and imbalanced oxidoreduction on the cellular membrane 4. This process has distinct morphological, mechanistical and genetic features that differ from those of apoptosis, necroptosis, pyroptosis and autophagy. Morphologically, ferroptosis is characterized by shrunken mitochondria, increased cell membrane density and a decreased number of mitochondrial cristae, which is consistent with changes in nuclear morphology and membrane integrity 8,9. In addition, whether cells undergo ferroptosis primarily depends on antagonism between the executive and defensive systems. Ferroptosis occurs when the accumulation of reactive oxygen species (ROS) overrides the antioxidant capacity provided by the antioxidant defensive system 10. Usually, multiple processes instigate ferroptosis, including iron metabolism, PUFA-PL synthesis and hydroxyl radical generation, which are collectively known as prerequisites for ferroptosis. In contrast, defensive systems, including the GPX4-dependent and the GPX4-independent defensive systems, play pivotal roles in suppressing ferroptosis by trapping lipid peroxyl radicals and neutralizing peroxidative reactions.

## Mechanism of Ferroptosis

Ferroptosis is characterized by precisely regulated molecular and signalling machineries. Mechanistically, lipid peroxides execute the ferroptosis process, which involves the interaction between the generation of oxidized phospholipids and the suppression of ferroptosis mainly through glutathione (GSH)-dependent and GSH-independent mechanisms. In this section, we summarize the current understanding of the regulatory networks involved in ferroptosis.

### Prerequisites of Ferroptosis

#### Labile iron pool (LIP)

Over the past few years, the field of iron metabolism has gained momentum due to increased recognition of cellular processes, including DNA synthesis, mitochondrial metabolism and cell proliferation. Iron is also a double-edged sword 11. The main source of iron is the breakdown of senescent erythrocytes after phagocytosis by macrophages, while a small proportion of iron is absorbed through the intestinal tract from food. Generally, plasma iron is mainly absorbed in the form of divalent iron (Fe^2+^) in duodenal enterocytes 12. Divalent iron is oxidized into trivalent iron by ceruloplasmin and then transported to various tissues and cells bound to transferrin 13. Inorganic trivalent iron (Fe^3+^) accompanied by transferrin binds to transferrin receptor 1 (TFR1) on the cellular membrane 14 and is subsequently released into the cytoplasm^15^. Subsequently, Fe^3+^ is reduced back to Fe^2+^ by the six-segment transmembrane epithelial antigen of prostate 3 (STEAP3) 16, is released into the cytosol by divalent metal transporter 1 (DMT1) and is ultimately stored in the labile iron pool (LIP) 17.

The linchpin involved in ferroptosis is iron deposition 18, and an imbalance in iron homeostasis may trigger ferroptotic cell death. Normally, intracellular iron is balanced in the form of LIP through well-established regulation of iron metabolism, including iron uptake, utilization, storage and release, due to the lack of effective iron excretion mechanisms in the organism 18. Imbalanced LIPs can induce or inhibit ferroptosis depending on whether the level of iron increases or decreases, respectively. Accumulating evidence has revealed that iron metabolism governs ferroptosis in a variety of ways. On the one hand, excessive iron is toxic and initiates the nonenzymatic Fenton reaction, generating hydroxide and hydroxyl radicals, which can subsequently induce ferroptosis 19,20; on the other hand, iron can act as a prominent catalyst for enzymes such as cytochrome P450 oxidoreductase (POR) and arachidonate lipoxygenase (ALOX), which participate in lipid peroxidation 21-25. LIP dysfunction can be rescued by iron chelators. Conversely, the suppression of iron chelators to increase susceptibility to ferroptosis could be a promising therapeutic strategy for cancer treatment. H-Ferritin, a nontoxic form of stored iron, suppresses hepatoma cell sensitivity to RSL3-induced ferroptosis 26.

#### PUFA-PL synthesis and peroxidation

**Figure 1:**
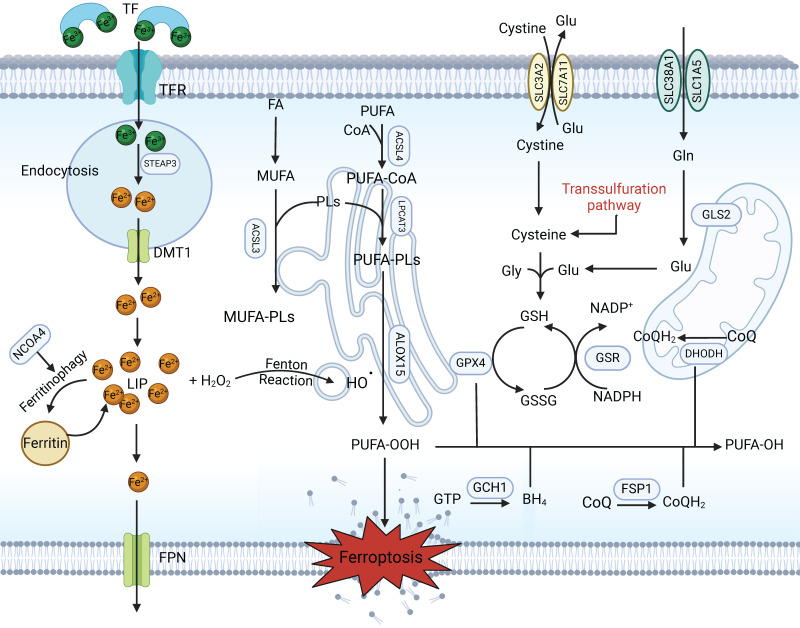
** Canonical mechanism of ferroptosis.** Ferroptosis is a type of regulated cell death characterized by excess iron in the LIP and the lethal accumulation of lipid peroxidation products. Fe^3+^ is transferred into the cell by TFR1, is converted to Fe^2+^ in the endosome and is then released from the endosome by DMT1. Iron-containing ferritin is degraded to produce a large amount of free iron via NCOA4-mediated ferritinophagy, resulting in ferroptosis. The Fenton reaction promotes lipid peroxidation by activating lipoxygenases. Cystine is taken up by System Xc^-^ for the synthesis of GSH, which further enhances the anti-lipid peroxidation activity of GPX4. In addition, FSP1-CoQ10 and GCH1-BH4 are two parallel GSH-independent pathways involved in the suppression of ferroptosis.

Ferroptosis is characterized by excessive peroxidation of polyunsaturated fatty acid-containing phospholipids (PUFA-PLs), which are toxic to cellular membranes. The accumulation of lipid peroxides is recognized as a determinant of ferroptosis. PUFA-PL synthesis and peroxidation involve the participation of several rate-limiting enzymes, including acyl-coenzyme A (CoA) synthetase long chain family member 4 (ACSL4) and lysophosphatidylcholine acyltransferase 3 (LPCAT3). PUFAs, such as arachidonic acid and adrenic acid, are ligated to CoA under the catalysis of ACSL4 to produce PUFA-CoA, which is subsequently re-esterified and incorporated into PLs by LPCAT3 to form PUFA-PLs 27,28 **(Figure 1)**. PUFA-PLs are particularly vulnerable to peroxide not only in the presence of bis-allylic moieties in PUFAs 19 but also in the presence of oxygen radicals and ROS stemming from hydrogen peroxide through the Fenton reaction. Eventually, PUFA-PLs disrupt the lipid bilayer membranes, which leads to dysfunction and ferroptosis. Recent findings also revealed that peroxisome-mediated plasmalogen biosynthesis provides another source of PUFAs for lipid peroxidation 29. In addition, exogenous monounsaturated fatty acids (MUFAs) can block ROS accumulation and suppress ferroptosis, which is characterized by reduced total levels of PUFA-PLs at the plasma membrane, in an ACSL3-dependent manner 30.

Within this context, ACSL4 is central to this process. ACSL4 was identified by a number of independent studies to be a key metabolic determinant in sensitizing cells to ferroptosis through the selective enrichment of PUFAs, more specifically arachidonic acid (AA)-containing species 28,31. Ectopic ACSL4 promotes or attenuates ferroptosis. Studies have revealed that PKCβII, a member of the protein kinase C (PKC) family that acts as a sensor of lipid peroxidation, amplifies lipid peroxidation by phosphorylating and activating ACSL4 and is involved in the lipid peroxidation-PKCβII-ACSL4 positive feedback axis, which promotes ferroptosis onset 32. Wang et al. demonstrated that IFNγ secreted by cytotoxic T lymphocytes (CTLs) and AA coordinately induces ferroptosis via ACSL4 in cancer cells 33. Interestingly, in a novel study, researchers reported that ACSL4 deficiency prevents ferroptotic cell death in hepatocytes but does not aggravate tumour progression and instead results in less fibrosis and proliferation. Hence, ACSL4-dependent processes have an unanticipated cancer-promoting effect on HCC tumorigenesis 34. Therefore, further therapies aimed at increasing ferroptosis sensitivity could consider the cancer-promoting function of ACSL4. In addition, the levels of lipid peroxides can be enhanced enzymatically by the lipoxygenase family (including ALOXE3, ALOX5, ALOX12, and ALOX15) in mammalian cells 35,36.

### Ferroptosis Defensive Systems

#### GSH-dependent defensive system

Glutathione (GSH), the most abundant intracellular antioxidant, is a thiol-containing tripeptide derived from three amino acids (glycine, glutamate and cysteine), with cysteine being the rate-limiting cofactor and precursor 37-40. GSH plays an essential role in maintaining redox homeostasis and neutralizing ROS to limit ferroptosis and serves as the substrate of choice for glutathione peroxidase 4 (GPX4), which is capable of detoxifying lipid peroxides into lipid alcohols 41,42. GSH deficiency or depletion directly triggers ferroptotic cell death. De novo synthesis of GSH depends on cysteine obtained through cystine (an oxidized dimer of cysteine) uptake through a cystine/glutamate exchange transporter, known as System Xc^-^; this transporter consists of two subunits, solute carrier family 7 member 11 (SLC7A11) and solute carrier family 3 member 2 (SLC3A2) 43,44 **(Figure 1)**. Therefore, ferroptosis can be potently induced by cysteine deprivation and GPX4 inhibition. Small pharmacological inhibitors, including the GPX4 inhibitor RSL3, erastin and sorafenib, which are direct inhibitors of the System Xc^-^-mediated import function, are widely used for the induction of ferroptosis 4,45.

It is believed that the SLC7A11-GSH-GPX4 axis constitutes the major cellular system that defends against ferroptosis. Dysfunction of this axis can affect the occurrence of ferroptosis. Mounting research has been devoted to examining and clarifying the roles and mechanisms of the ferroptosis defence system in tumour suppression. Some tumour suppressors, including p53 and IFNγ, can hamper SLC7A11 expression and transport activity 46,47. In contrast, tumour cells take advantage of stress-related transcription factors, including nuclear factor erythroid 2-related factor 2 (NRF2) and activating transcription factor 4 (ATF4), to increase SLC7A11 expression to combat ferroptosis. Some factors, such as 8 open reading frame 76 (C8orf76) and the RNA-binding protein DAZAP1, have been shown to inhibit ferroptosis through increasing the transcription of SLC7A11 in HCC 48,49. Moreover, enhancing the ubiquitination-mediated degradation of SLC7A11 to induce ferroptosis by increasing the expression of suppressor of cytokine signalling 2 (SOCS2) may enhance the efficacy of HCC radiotherapy and improve patient prognosis 50. Furthermore, many studies have demonstrated that GSH depletion can reduce lipid peroxide levels and that targeting GSH to decrease oxidative stress can suppress ferroptosis. Further research revealed that TGF-β1/Smad3 signalling plays a role in the repression of SLC7A11 activity and that promoting lipid peroxidation increases vulnerability to GPX4 inhibition in HCC cells 51.

However, some cancer cells remain resistant to ferroptosis in the absence of GPX4 activation 52, which suggests the existence of alternative mechanisms to counter cellular oxidative stress and suppress ferroptosis.

#### GSH-independent defensive system

Promisingly, ferroptosis suppressor protein 1 (FSP1) has been identified as a potential endogenous ferroptosis suppressor that functions in parallel with GPX4 to confer ferroptosis resistance 53,54. FSP1, which is a second mainstay of ferroptosis control after GPX4^55^, is also known as flavoprotein apoptosis-inducing factor mitochondria-associated 2 (AIFM2). The protein encoded by this gene is located on the plasma membrane, where it suppresses lipid hydroperoxides by catalysing the reduction of ubiquinone (CoQ)^56^,^57^ back to ubiquinol (CoQH2)^54^, which can trap lipid peroxyl radicals, thereby blocking PUFA-PL synthesis and ferroptosis. The expression of FSP1 is positively associated with the resistance of cells to GPX4 inhibitors and is essential for maintaining tumour cell growth in the absence of GPX4^54^. Therefore, FSP1 is a potent antioxidant that contributes to ferroptosis resistance 58. A recent study identified FSP1 as a novel, vulnerable therapeutic target in HCC 59. Furthermore, disrupting FSP1 is a promising therapeutic approach for HCC patients with high-density lipoprotein-binding protein (HDLBP) or lncFAL expression 60.

In addition, another antiferroptosis system is composed of dihydroorotate dehydrogenase (DHODH), which mechanistically operates in parallel with mitochondrial GPX4 to detoxify lipid peroxidation products and block ferroptosis by reducing CoQ to CoQH2^61^. Unlike FSP1, DHODH is localized to the inner mitochondrial membrane. DHODH has been intensively explored in recent years as a promising target for cancer therapy 62,63. It has been proposed that overexpression of the oncogenic protein ubiquitin-conjugating enzyme E2T (UBE2T) in HCC leads to the upregulation of DHODH, thereby promoting HCC development 64.

Cancer cells rewire their metabolism and rely on endogenous antioxidants to mitigate lethal oxidative damage to lipids. Recently, a novel pathway named GTP cyclohydrolase 1 (GCH1)-tetrahydrobiopterin (BH4) was reported to be an essential metabolic signalling pathway upon GPX4 inhibition 65,66. Mechanistically, BH4 is a potent radical-trapping antioxidant that protects lipid membranes from autoxidation, both alone and in combination with vitamin E 67,68. Early reports have verified that GCH1, which originates from GTP, is a governing rate-limiting enzyme in the synthesis of BH4 and has the outstanding capacity to eliminate lipid peroxidation and prevent RSL3-induced ferroptosis 67,69. GCH1 overexpression exhibits robust protection against RSL3- and IKE-induced ferroptosis and genetic ablation of GPX4-induced ferroptosis but does not protect cells against inducers of apoptosis and is only marginally effective against necroptosis, which indicates that GCH1 selectively counters ferroptotic cell death 65. Thus, these results indicate that the GCH1-BH4 pathway acts as an endogenous antioxidant pathway to inhibit ferroptosis through a mechanism independent of the glutathione system. Although studies on the GCH1-BH4 pathway have not been applied to the inhibition of ferroptosis in HCC, the GCH1-BH4 pathway can undoubtedly promote HCC progression 70.

## Ferroptosis in the Hepatocellular Carcinoma-associated Signalling Pathway

### Noncoding RNA

**Figure 2:**
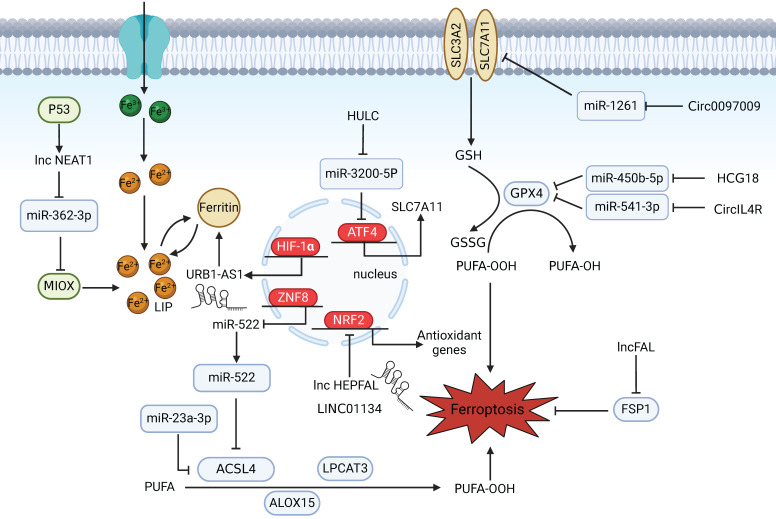
** Noncoding RNAs participate in regulating ferroptosis in HCC.** NEAT1 indirectly upregulates MIOX expression by competitively binding to miR-362-3p, thereby promoting erastin- and RSL3-induced ferroptosis in HCC cells. p53 can increase NEAT1 expression. URB1-AS1 mitigates sorafenib-induced ferroptosis by inducing ferritin phase separation and reducing the free iron content. HIF-1α promotes URB1-AS1 expression. The ZNF8/miR-552-5p/ACSL4 and ETS1/miR-23a-3p/ACSL4 axes regulate sensitivity to ferroptosis. LncRNA HEPFAL promotes ferroptosis by modifying SLC7A11 ubiquitination. LINC01134 positively regulates GPX4 through NRF2. Downregulation of HULC induces ferroptosis in liver cancer cells by targeting the miR-3200-5p/ATF4 axis to modulate HCC development. Circ0097009 acts as a ceRNA to regulate the expression of SLC7A11 by sponging miR-1261. The lncRNAs HCG18 and CircIL4R sponge miR-450b-5p and miR-541-3p, respectively, to regulate GPX4. lncFAL reduces vulnerability to ferroptosis by binding to FSP1.

As a result of the exponential growth in ferroptosis research, the participation of ncRNAs in regulating ferroptosis in HCC has also been reported. Mechanistically, miRNAs function by binding to complementary sequences in key genes involved in ferroptosis and suppressing their expression 71,72** (Figure 2)**. In one study, miR-23a-3p acted as a direct suppressor of ferroptosis by targeting the 3′‑untranslated region (UTR) of ACSL4 and was found to be responsible for the acquisition of sorafenib resistance in sorafenib‑treated HCC cells 73. The mechanisms by which lncRNAs and circRNAs govern gene expression are relatively complex. On the one hand, they can act as ceRNAs to sponge miRNAs to block mRNA degradation in ferroptotic cell death and regulate the binding of transcription factors to promoters 74. For example, as ceRNAs, HCG18 and CircIL4R can sponge miR-450b-5p and miR-541-3p, respectively, to modulate the expression of GPX4 and inhibit ferroptosis in HCC 75,76. Similarly, circ0097009 acts as a competing endogenous RNA that regulates the expression of SLC7A11 by sponging miR-1261 in HCC 77. Furthermore, the long noncoding RNA NEAT1 is upregulated in HCC cells after erastin and RSL3 treatment and cooperates with miR-362-3p and its downstream Myo-inositol oxygenase (MIOX), a nonheme ferritin, to form a ceRNA network that can eventually promote ferroptosis 78. On the other hand, lncRNAs and circRNAs can also function as scaffolds to regulate protein-protein interactions and related downstream signalling pathways. In one study, LINC01134, a promising lncRNA, promoted NRF2 recruitment to the GPX4 promoter region to transcriptionally regulate GPX4. Facilitating ferroptosis by knocking down LINC01134 could be a potential therapeutic strategy for the treatment of HCC 79. Recently, the ferroptosis-related lncRNA URB1-AS1, which is highly expressed in sorafenib-resistant HCC samples and mitigates sorafenib-induced ferroptosis by inducing ferritin phase separation and reducing the cellular free iron content, was identified through lncRNA sequencing. Silencing URB1-AS1 successfully enhanced the sensitivity of HCC cells to sorafenib in an in vivo tumour model 80.

Additionally, some studies have shown that ncRNAs participate in the epigenetic modulation of chromatin to regulate gene expression. Overall, ncRNAs may serve as diagnostic biomarkers for HCC and as potential targets for HCC therapy.

### NRF2

**Figure 3:**
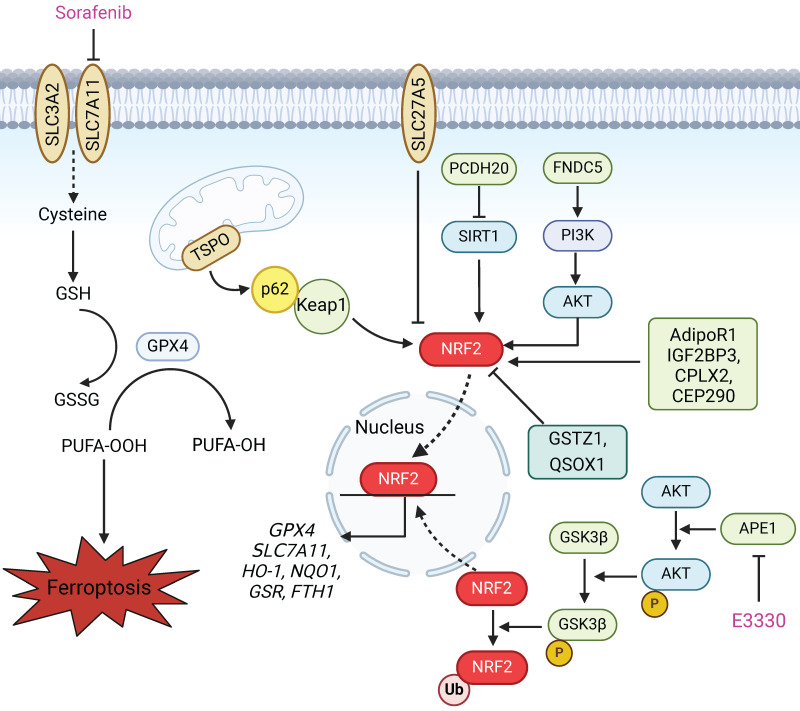
** NRF2 is involved in preventing lipid peroxidation and ferroptosis in HCC.** Upon exposure of HCC cells to specific ferroptosis inducers, such as sorafenib, erastin and RSL3, the expression of P62 increases, which prevents degradation of the NRF2 protein by competitively binding to the Keap1 protein. This process also promotes the entry of NRF2 into the nucleus to initiate the transcription of downstream ferroptosis-related proteins, including GPX4, SLC7A11, HO-1, NQO1, GSR and FTH1. Many studies have shown that various proteins affect the sensitivity of HCC cells to ferroptosis via the NRF2 pathway. TSPO inhibits ferroptosis in HCC cells through the P62/KEAP1/NRF2 antioxidant pathway. SLC27A5 inhibits the NRF2/GSR pathway to reduce GSH in sorafenib-sensitive HCC cells. PCDH20 promotes ferroptosis by suppressing the expression of SIRT1 and thus promoting NRF2 acetylation in HCC. FNDC5 activates NRF2 through the PI3K/AKT pathway, conferring resistance to ferroptosis. AdipoR1, IGF2BP3, CPLX2 and CEP290 inhibit ferroptosis in liver cancer cells by activating the NRF2 pathway. GSTZ1 and QSOX1 enhance the sensitivity of HCC cells to sorafenib-induced ferroptosis via inhibition of the NRF2 pathway. Furthermore, APE1 inhibition promotes the degradation of NRF2 through the AKT/GSK3β-mediated ubiquitin‒proteasome pathway and subsequently suppresses the NRF2/SLC7A11/GPX4 axis, thereby facilitating ferroptosis.

Nuclear factor erythroid 2-related factor 2 (NRF2) is considered a master regulator of the antioxidant response. NRF2 can translocate to the nucleus to initiate the transcription of antioxidant response element (ARE)-containing genes, which are involved in preventing or correcting redox imbalances in the cell in different contexts **(Figure 3)**. Notably, two inducers of ferroptosis, RSL3 and erastin, initiate the ferroptotic cascade by inhibiting glutathione GPX4 and SLC7A11, both of which are downstream targets of NRF2. Furthermore, many other proteins and enzymes associated with glutathione biosynthesis, the antioxidant response, and lipid and iron metabolism, such as HO-1, NQO1, GSR, FTH1, FTL, and ferroportin, which are responsible for preventing lipid peroxidation and thus causing ferroptosis, are target genes of NRF2^81^. Therefore, ferroptotic cell death is largely associated with NRF2 dysfunction. NRF2 status is a key factor that determines the therapeutic response to ferroptosis-targeted therapies and improves resistance to chemotherapeutic drugs in HCC cells 82. Several studies have shown that the NRF2 status is modulated by a variety of proteins and drugs, which in turn affects the occurrence of ferroptosis in HCC (Figure 3). According to recent research, the mitochondrial translocator protein (TSPO), which is involved in a broad range of mitochondrial functions, inhibits ferroptosis in HCC cells through the P62/KEAP1/NRF2 antioxidant pathway 83. Protocadherin 20 (PCDH20) can promote ferroptosis by preventing Sirtuin 1 (SIRT1) from deacetylating NRF2, which leads to the downregulation of SLC7A11, GPX4, and GSH in HCC 84. In addition, ABCC5, a member of the ATP-binding cassette (ABC) transporter family, and fibronectin type III domain containing 5 (FNDC5) can activate NRF2 through the PI3K/AKT pathway, which confers resistance to ferroptosis and reduces the anticancer activity of sorafenib in HCC cells 85,86. Tiliroside, a potent TANK-binding kinase 1 (TBK1) inhibitor, induces ferroptosis via the P62/KEAP1/NRF2 pathway and eventually increases the sensitivity of HCC cells to sorafenib 87. A recent study reported that apurinic/apyrimidinic endonuclease 1 (APE1), a key enzyme with dual functions in DNA repair and redox regulation, enhances ferroptosis through regulation of the NRF2/SLC7A11/GPX4 axis in HCC. Research on tumour xenografts in nude mice further demonstrated that the inhibition of APE1 blocks HCC progression and contributes to ferroptosis-based HCC therapy 88. Thus, the induction of lipid peroxidation and ferroptosis by regulating the NRF2 pathway is a promising strategy for enhancing the anticancer effects of chemotherapy and radiotherapy, especially in therapy-resistant HCC cells.

### HIF-1α

Given that an imbalance between insufficient blood supply and the high oxygen consumption needed to support the rapid growth of tumour cells leads to widespread hypoxia in the tumour microenvironment (TME), hypoxia tightly regulates tumour cell apoptosis, chemoresistance, invasion, immune escape, and many other biological functions 89-91. In addition, hypoxia is accompanied by the production of ROS, which trigger oxidative stress 92. Hypoxia-inducible factor 1 alpha (HIF-1α) is a crucial regulator of the response to hypoxic stress in cells. Several pioneering studies have shown that HIF-1α is an important driver of susceptibility to ferroptosis in cancer cells, including HCC cells, by modulating the transcription of numerous genes involved in iron metabolism, lipid metabolism, glycolysis and glutamate metabolism. Recently, evidence has suggested that HIF-1α serves as the main driver of ferroptosis resistance in tumour cells under hypoxic conditions through the generation of a lactate-induced acidic environment and enhancement of the transcription of the glutamate transporter SLC1A1^93^. In HCC, copper metabolism MURR1 domain 10 (COMMD10) inhibits the HIF-1α/CP loop, which enhances ferroptosis and radiosensitivity by disrupting Cu-Fe homeostasis 94. Moreover, lipid metabolism reprogramming steered by HIF-1α results in a reduction in the synthesis of polyunsaturated fatty acids (PUFAs), which are substrates of lipid peroxidation, and thus reduces sensitivity to ferroptosis. Recently, FASN, an enzyme that regulates the de novo synthesis of fatty acids, was shown to bind to and upregulate HIF-1α and its downstream target SLC7A11 to inhibit HCC ferroptosis and promote sorafenib resistance 95. Nevertheless, the potential of HIF-1α as a therapeutic target requires further research. The complex relationship between HIF-1α expression and various factors, including the mutational landscape of HCC, the tumour microenvironment, and the administration of other therapeutic agents, requires comprehensive consideration. In conclusion, understanding the links between HIF-1α and ferroptosis provides a novel perspective for understanding HCC pathology and may offer potential novel treatment strategies.

### p53

The tumour suppressor p53 is considered the guardian of the genome and participates in multifaceted cellular activities, including cell cycle arrest, senescence, and apoptosis 96. Remarkably, numerous studies have provided evidence that p53, a hub involved in cellular redox regulation and a therapeutic target in cancer, has various effects on ferroptotic cell death 97. On the one hand, p53 has been shown to increase sensitivity to ferroptosis through the regulation of multiple downstream targets through either transcriptional or posttranslational mechanisms. First, p53 can decrease the expression of SLC7A11, which is necessary for the uptake of cystine and may contribute to tumour suppression in vitro and in vivo 46. Second, the induction of glutaminase 2 (GLS2) and spermidine/spermine N1-acetyltransferase 1 (SAT1) at the transcriptional level promotes ferroptosis, favouring glutaminolysis and the formation of lipid peroxides, respectively 98,99. Dysfunctional p53 signalling is one of the major causes of HCC tumorigenesis and development. Research has shown that other components of the p53 network can also regulate ferroptosis in HCC. For example, p53 can activate ALOX12, a lipoxygenase, indirectly by transcriptional repression of SLC7A11, which results in ALOX12-dependent ferroptosis upon ROS stress and alleviation of tumorigenesis 35. The protocadherin gene PCDHB14, a novel p53 target gene, promotes ferroptosis to inhibit SLC7A11 expression via the NF-κB signalling pathway in HCC 100. Another study revealed that Krüppel-associated box (KRAB)-type zinc-finger protein (ZNF498) functions as an oncogene in promoting HCC carcinogenesis by suppressing ferroptosis via interactions with p53, which decreases p53 Ser46 phosphorylation 101. In addition, a study revealed that Gls2 KO mice have a marked propensity to develop late HCC, which suggests that GLS2 plays a role in ferroptosis and consequent tumour suppression 102. On the other hand, p53 suppresses ferroptosis through direct inhibition of dipeptidyl peptidase 4 (DPP4) activity 103. By identifying a new p53 acetylation site at lysine K136, mutations at all five acetylation sites (p53-5KR) completely/greatly reduced the remaining tumour suppressive function of p53^104^,^105^, which indicates that p53 acetylation plays an important role in regulating ferroptosis and inhibiting tumour development. Given that p53 can modulate multiple target genes involved in various biological processes, the exact role of p53 in ferroptosis is likely to depend on the specific context.

### Metabolic Dysregulation

**Figure 4:**
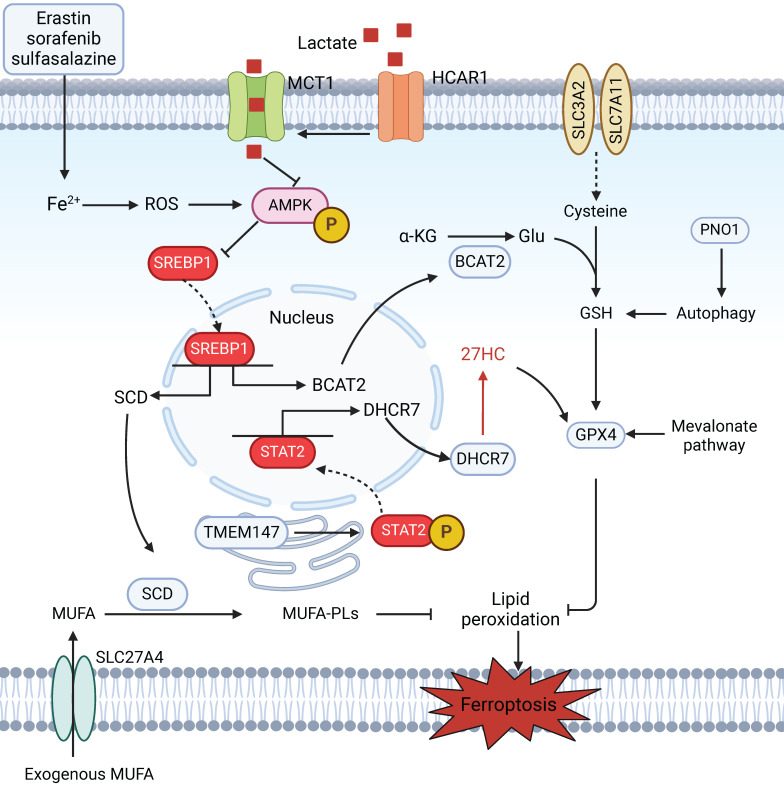
** Metabolic dysregulation mediates ferroptosis in HCC.** The AMPK/SREBP1 pathway plays a pivotal role in mediating metabolic homeostasis during ferroptosis. HCAR1/MCT1-mediated lactate uptake promotes ATP production in HCC cells and deactivates AMPK, which leads to the upregulation of SREBP1 and downstream SCD1 to enhance the production of MUFAs; this results in resistance to lipid peroxidation and ferroptosis in HCC cells. In addition, SLC27A4 overexpression promotes the selective uptake of MUFAs in HCC cells. BCAT2 is the key enzyme that regulates intracellular glutamate levels and is a specific inhibitor of ferroptosis. Ferroptosis inducers (erastin, sorafenib, and sulfasalazine) can activate the AMPK/SREBP1 pathway and subsequently inhibit BCAT2 transcription. Moreover, the level of intracellular glutamate could be promoted by PNO1-induced autophagy, which results in the accumulation of GSH and ferroptosis in HCC. The mevalonate pathway affects the synthesis of CoQ and GPX4 by regulating the maturation of selenocysteine tRNA. Increased levels of 27HC also upregulate GPX4 in HCC, leading to ferroptosis resistance via the TMEM147/STAT2/DHCR7 axis.

Accumulating evidence indicates that cellular metabolism plays a crucial role in ferroptosis 106,107. Intracellular iron metabolism is essential for ferroptosis through either iron-dependent oxidases or the Fenton reaction. A recent report suggested that the autophagic degradation of ferritin regulates ferroptosis through the autophagy cargo receptor nuclear receptor coactivator 4 (NCOA4) 108. Amino acid metabolism also influences the susceptibility of HCC cells to ferroptosis. Branched-chain amino acid aminotransferase 2 (BCAT2) suppresses ferroptosis by mediating the metabolism of sulfur-containing amino acids and regulating intracellular glutamate levels 109 **(Figure 4)**. Additionally, the mevalonate (MVA) pathway not only affects the synthesis of CoQ and GPX4, two key regulators of ferroptosis, by regulating the maturation of selenocysteine tRNA, but also provides several antioxidant intermediates, such as squalene and 7-dehydrocholesterol, which act as radical-trapping agents to suppress lipid peroxidation 110,111. According to recent reports, the RNA-binding protein partner of NOB1 (PNO1) protects HCC cells from ferroptotic cell death by increasing the synthesis and accumulation of intracellular glutamate 112. The liver, the central organ of fatty acid metabolism, is enriched with a diverse range of lipids 113. Interestingly, the occurrence and development of ferroptosis are closely related to lipid metabolism. Aberrant lipid metabolism, such as enhanced the cellular uptake of exogenous PUFAs via the overexpression of the fatty acid transporter enzyme CD36, could increase the cellular PUFA-PL content, thereby promoting lipid peroxidation and ferroptosis. In contrast, treatment with exogenous MUFAs results in a reduction in the amount of PUFA-PLs in cell membranes and inhibition of lipid peroxidation and subsequent ferroptosis 30. In one study, solute carrier family 27 member 4 (SLC27A4), a fatty acid transporter protein, promoted the selective uptake of MUFAs, which resulted in a reduction in lipid peroxidation and resistance to ferroptosis 114. In addition, elevated cholesterol levels in the tumour microenvironment (TME) are a common feature of various cancers. A metabolic derivative of cholesterol, 27-hydroxycholesterol, has been reported to trigger ferroptosis resistance by chronic selection of cancer cells with increased uptake and/or lipid biosynthesis, including cholesterol 115; other studies have shown that the protective role of cholesterol is ascribed to its ability to decrease membrane fluidity and promote lipid raft formation, which affects the diffusion of LPO substrates and subsequent resistance to ferroptosis 116. Therefore, future research should aim to determine additional connections between metabolic dysregulation and ferroptosis in HCC, which will pave the way for more effective therapeutic strategies.

## Ferroptosis in Hepatocellular Carcinoma Therapy

### Chemotherapy

Patients with advanced HCC generally have fewer opportunities for surgery, and interventions such as interventional therapies are often limited due to liver function and other reasons. Therefore, targeted therapy is usually the main treatment option, and it is popular because of its minimal side effects, good efficacy, and convenience. Currently, three main targeted drugs are used to treat advanced HCC in China. Among them, first-line treatments include sorafenib and lenvatinib, while second-line treatment includes regorafenib.

**Figure 5:**
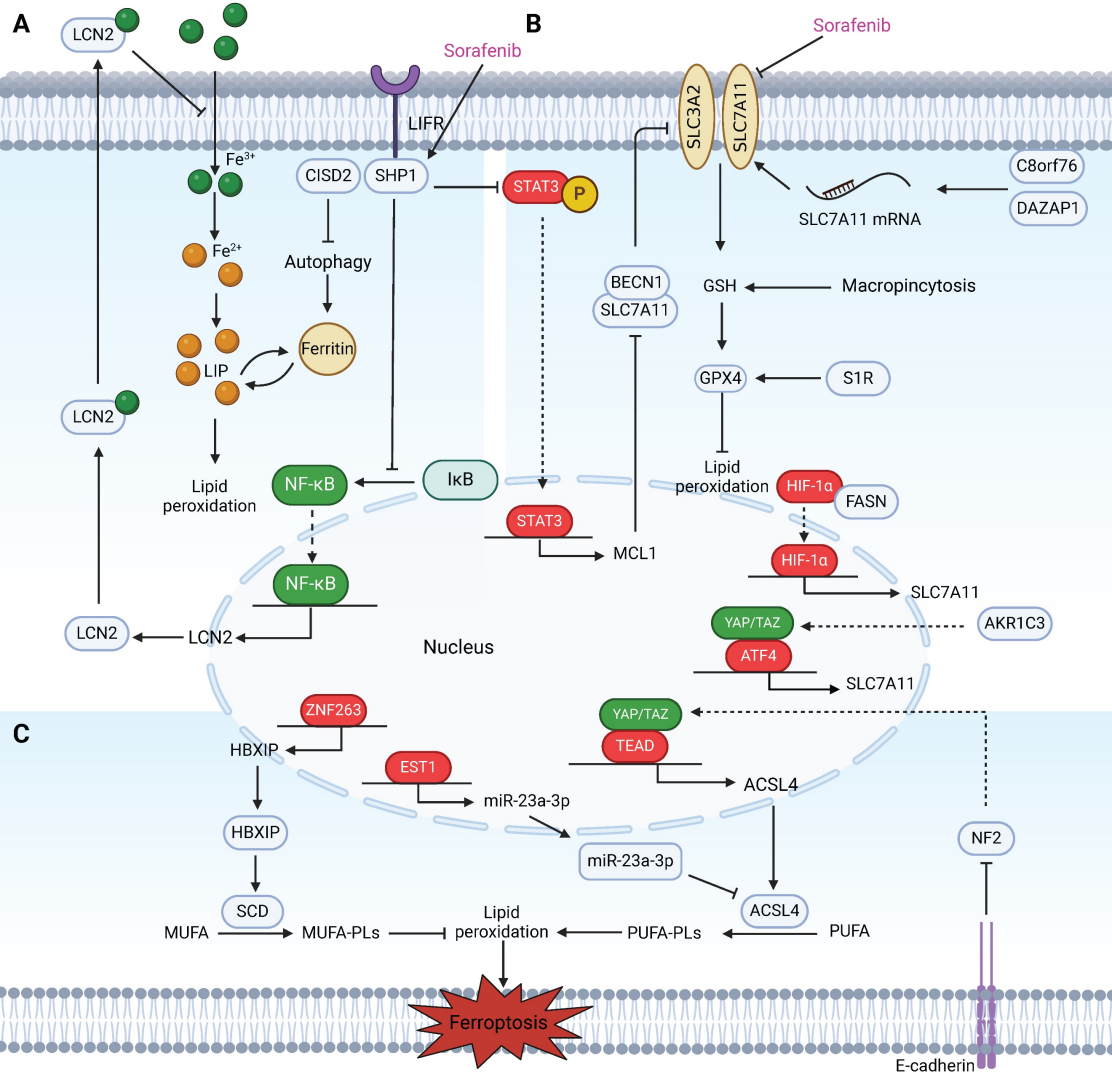
** Regulatory factors involved in sensitivity and resistance to ferroptosis in HCC patients treated with sorafenib.** These regulatory factors mainly participate in sensitivity and resistance to ferroptosis in HCC cells treated with sorafenib through three pathways. (A) Labile iron pool: Inhibition of CISD2 promotes excessive iron accumulation through autophagy, resulting in sorafenib-induced ferroptosis in resistant cells. LIFR sensitizes HCC cells to sorafenib-induced ferroptosis through NF-κB inhibition and the subsequent downregulation of iron-sequestering LCN2. (B) The biosynthesis of PUFAs: Activation of the HBXIP/SCD axis via coactivation of ZNF263 reduces the anticancer activity of sorafenib and suppresses ferroptosis. MiR-23a-3p acts as a direct suppressor of ferroptosis by targeting the 3'UTR of ACSL4. The ETS1/miR‑23a‑3p/ACSL4 axis contributes to sorafenib resistance in HCC by regulating ferroptosis. Inhibition of the Hippo signalling pathway can activate YAP to promote the transcription of ACSL4, thereby promoting ferroptosis. The interaction mediated by E-cadherin in HepG2 cells suppresses ferroptosis by activating the intracellular NF2 and Hippo signalling pathways. (C) The defensive system against ferroptosis: In sorafenib-resistant HCC cells, YAP/TAZ and ATF4 are activated in the nucleus where they induce SLC7A11 expression. AKR1C3 suppresses ferroptosis through the regulation of YAP/SLC7A11. Binding between BECN1 and SLC7A11 increases, which inhibits the activity of System Xc^-^ and the triggering of ferroptosis in sorafenib-treated HCC cells via the SHP-1/STAT3/MCL1 axis. C8orf76 and DAZAP1 reduce cellular sensitivity to sorafenib by acting on SLC7A11 through different mechanisms. Additionally, targeting the FASN/HIF1α/SLC7A11 pathway could resensitize HCC cells to sorafenib. S1R and macropinocytosis negatively regulate sorafenib-induced ferroptosis.

Sorafenib is a multitarget, multikinase inhibitor and first-line molecular-target drug for the treatment of advanced HCC that prolongs the median overall survival to 6.5 months 117. Ferroptosis is essential for sorafenib-induced cell death and has been implicated in the pathology of HCC; thus, ferroptosis induction is a promising novel cancer treatment 118,119. Notably, sorafenib has been reported to block SLC7A11 function, subsequent ROS accumulation and GSH depletion, in addition to its well-known inhibition of many angiogenesis-associated kinases (e.g., VEGFR, FGFR, PDGFR) 120. Thus, it is reasonable to speculate that genetic alterations involved in ferroptosis may regulate sorafenib sensitivity and mediate drug resistance during the development or treatment of HCC, which adds to the complexity of the antitumour mechanism of sorafenib** (Figure 5)**. A few studies have explored this hypothesis. YAP/TAZ 121 and C8orf76^48^ act as negative regulators of ferroptosis by upregulating SLC7A11 transcription, thus driving resistance to sorafenib in hepatocellular carcinoma. Louandre C et al. reported that, upon exposure to sorafenib, the reduced levels of Rb achieved through stable RNA interference in HCC cells promote the onset of ferroptosis 122. In one study, the positive-acting enzyme ACSL4, whose expression is independent of sorafenib treatment in HCC cell lines, can serve as a promising predictive and validated biomarker that contributes to the precise treatment of HCC that is superior to that involving Rb 123. In addition, the ability of sorafenib to regulate a series of downstream effectors, such as MT1 and HBXIP, increases sensitivity to ferroptosis 124,125. Apart from some research already devoted to deciphering the mechanisms that enhance sorafenib-induced ferroptosis, it is imperative to identify resistance targets and combination drugs, which are essential for overcoming the dilemma of sorafenib resistance due to their limited survival. Here, we summarize some compounds that can target other essential aspects of ferroptosis that may synergize with sorafenib to inhibit HCC **(Table 1)** and compounds or drugs that trigger ferroptotic cell death that exhibit antitumour effects independent of sorafenib-induced ferroptosis **(Table 2)**.

In recent years, the new targeted therapy drug lenvatinib has shown good efficacy, especially in the treatment of HBV-related HCC, and has become the second first-line drug for the treatment of advanced liver cancer after sorafenib. Compared with sorafenib, lenvatinib significantly improved the objective response rate (ORR), progression-free survival (PFS) and time to progression (TTP) in one study 126. This finding demonstrated the superior antitumour effect of lenvatinib over sorafenib.

Lenvatinib has been approved as an alternative option for patients who do not respond to sorafenib treatment, and its targets include VEGFR, PDGFR, FGFR1/2/3/4, KIT, and RET 127-130. An association between ferroptosis and lenvatinib has also been reported. One study revealed that lenvatinib suppressed SLC7A11 and GPX4 expression, which resulted in the accumulation of lipid ROS in Hep3B and HuH7 cells. These findings further confirmed that lenvatinib induces ferroptosis by inhibiting FGFR4. FGFR4 expression in cancer cells is related to the therapeutic efficacy of lenvatinib in patients with HCC. The PFS of FGFR4-positive HCC patients is longer than that of FGFR4-negative patients. Additionally, NRF2 is involved in the regulation of this process 131. As with sorafenib, resistance to lenvatinib is common. Therefore, the exploration of the potential mechanisms of lenvatinib resistance in HCC is urgently needed. A recent study revealed that HAND2-AS1 promotes the expression of ferroptosis-related genes (TLR4, NOX2, and DUOX2) and promotes ferroptosis to reverse lenvatinib resistance in HepG2 lenvatinib-resistant cells and xenograft models 132. Lenvatinib can induce ferroptosis, which is also involved in lenvatinib resistance, but related research on this topic is lacking. Therefore, further research is needed to determine whether induction of ferroptosis to reverse lenvatinib resistance is a promising treatment strategy.

Regorafenib, a second-line targeted therapy for HCC, is typically used for liver cancer patients to prolong patient survival after failure of first-line treatment 133. Regorafenib is a multitargeted tyrosine kinase inhibitor that can inhibit tumour cell proliferation, suppress tumour angiogenesis, and modulate the tumour microenvironment. This drug targets VEGFR, PDGFR, B/C-Raf, KIT, RET, and FGFR1/2 134. Currently, no published studies have revealed the connection between regorafenib and ferroptosis in HCC.

### Antitumour Immunotherapy

In recent years, breakthroughs have been achieved in the application of immunotherapy in HCC patients. Atezolizumab (an anti-PD-L1 antibody) in combination with bevacizumab (a VEGF antagonist) was approved for first-line treatment of advanced-stage HCC 135. However, more sufficient medical evidence is still needed for immunotherapy as a first-line treatment. Recent research has revealed that ferroptosis is a form of immunogenic cell death (ICD) and has a certain degree of crosstalk with immunotherapy compared with other types of cell death, including apoptosis, autophagy and necrosis 136. On the one hand, the ferroptotic pathway can reshape the tumour immune microenvironment and improve anti-PD1 immunotherapy in HCC. Using single-cell RNA sequencing, one study identified a differentially expressed gene, APOC1, which is overexpressed in tumour-associated macrophages (TAMs) in HCC tissues. APOC1 inhibition can promote the transition of M2 macrophages to M1 macrophages through the ferroptotic pathway 137. Furthermore, for the first time, a recent study revealed ferroptosis upstream of immune cell activation and demonstrated that hepatocyte ferroptosis due to genetic GPX4 ablation causes a complex immune response that includes triggering CD8+ T-cell recruitment and inducing IFN-γ secretion by CD8+ T cells, which enhances PD-L1 expression on tumour cells. Thus, a novel therapeutic option for the treatment of HCC involving the combination of withaferin A (WFA) (a ferroptosis inhibitor), α-PD-1 and SB225002 (a CXCR2 inhibitor) has been proposed 138. On the other hand, the immune response can trigger tumour cell ferroptosis. For example, in anti-PD-L1 immunotherapy, IFN-γ secreted by activated CD8+ T cells downregulates the expression of SLC3A2 and SLC7A11, impairs the uptake of cystine by tumour cells, and consequently promotes ferroptosis-specific lipid peroxidation in tumour cells 47. In addition, IFN-γ stimulates ACSL4 and alters the lipid profile of tumour cells, which promotes ACSL4-dependent tumour ferroptosis 139. Thus, cancer immune checkpoint blockade paired with selective fatty acids is a potential anticancer approach. However, studies have shown that CD8+ T cells lose their antitumour effector function in the TME when they take up fatty acids through CD36, which induces ferroptosis and results in decreased cytotoxic cytokine production by cells. Inhibition of CD36-mediated ferroptosis in combination with ICB greatly enhances the antitumour effects of CD8+ T cells 140. Therefore, to some extent, ferroptosis also suppresses antitumour immunity and promotes tumour growth.

Many studies have identified key molecules and drugs that can change the efficacy of immunotherapy in patients with HCC. FSP1 inhibitor (iFSP1) exerts a better effect than anti-PD-L1 in prolonging survival time, and the combination of iFSP1 and anti-PD-L1 can further inhibit the progression of HCC in mice 59. Phosphoglycerate mutase 1 (PGAM1) inhibition and the mitochondrial translocator protein (TSPO) inhibitor PK11195 exert antitumour effects by promoting ferroptosis, and these compounds can synergize with anti-PD-1 immunotherapy in HCC 83,141. Therefore, the addition of ferroptosis-based therapy to immunotherapy provides a new option for the personalized treatment of HCC patients. Overall, the intersection of ferroptosis and the immune system in HCC provides fertile ground for novel antitumour immunotherapeutic strategies, but the detailed mechanism underlying the relationship between ferroptosis and the TME is not fully understood, and extensive clinical research on how to induce ferroptosis safely and effectively in clinical practice is lacking.

## Future Perspectives and Conclusion

Ferroptosis is a novel form of programmed and nonapoptotic cell death driven by cellular metabolism and iron-dependent lipid peroxidation. Recent clarification of molecular pathways and regulatory proteins, such as GPX4, ACSL4, and NRF2, which control ferroptosis, has provided insight into the underlying mechanism of ferroptosis. However, we currently have only a patchy portrait of the importance of several genes involved in preventing or inducing ferroptosis, and several questions about the fine-tuning of ferroptotic cell death remain largely unanswered.

Hepatocellular carcinoma, one of the most prevalent malignancies and a leading cause of cancer-related mortality worldwide, is characterized by rapid disease onset and progression. With increasing research on ferroptosis, tremendous evidence indicates that ferroptosis plays a positive role in anti-HCC therapy and could become a promising therapeutic target. Therefore, this article focused on summarizing the hotspots and major findings in ferroptosis research in HCC, including the current understanding of ferroptosis mechanisms and research based on an increase in susceptibility to ferroptosis combined with chemotherapy or immunotherapy to treat HCC, which could improve anticancer effects.

Different viewpoints suggest that ferroptosis demonstrates unique advantages in its underlying mechanisms and implications for HCC therapy compared with other forms of cell death. Furthermore, ferroptosis has emerged as a potential therapeutic target for the treatment of HCC due to its distinct molecular pathways and vulnerability to pharmacological manipulation. Targeting key regulators of ferroptosis, such as GPX4 or the lipid peroxidation pathway, holds promise for selectively inducing cancer cell death. In contrast, conventional therapies for HCC, such as chemotherapy and radiotherapy, primarily induce apoptosis or necrosis and may not effectively target ferroptosis pathways. Immunotherapy, which harnesses the immune system to target cancer cells, has shown promising results in HCC treatment. Understanding the differences between ferroptosis and other forms of cell death is crucial for optimizing immunotherapeutic strategies. Ferroptosis may contribute to immunogenic cell death and enhance antitumour immune responses, but its interplay with immunotherapy requires further investigation. Finally, the combination of ferroptosis-inducing agents with immunotherapeutic approaches may offer synergistic benefits by promoting tumour cell death while stimulating antitumour immune responses.

In the application of ferroptosis therapy in HCC, treatment tolerance and responsiveness are critical factors that influence the effectiveness and safety of the therapy. Treatment tolerance mainly includes patient health status, the toxicity of ferroptosis inducers or inhibitors used to treat HCC, dosing and the risk of adverse effects of combining ferroptosis-based therapy with other treatment modalities, such as chemotherapy or immunotherapy. Furthermore, HCC is characterized by significant heterogeneity, both within individual tumours and among different patients. Some tumours may be more susceptible to ferroptosis induction due to specific molecular profiles, while others may be resistant. The tumour microenvironment, which includes factors such as hypoxia, inflammation, and stromal components, can modulate ferroptosis sensitivity. Strategies that modulate the tumour microenvironment to enhance treatment responsiveness need to be explored. The mechanisms underlying resistance to ferroptosis therapy, such as the upregulation of antioxidant pathways or alterations in iron metabolism, need to be understood and targeted to improve treatment responsiveness. Overall, achieving an optimal balance between treatment tolerance and treatment responsiveness is essential for the successful application of ferroptosis therapy in HCC. This requires a comprehensive understanding of the underlying mechanisms, patient-specific factors, and careful clinical management. Ongoing research aimed at elucidating these factors and optimizing treatment strategies will be critical for improving outcomes in HCC patients receiving ferroptosis-based therapy.

## Figures and Tables

**Table 1:** Agents that synergize with sorafenib.

Agents	Mechanism	Target	References
Carmustine (BCNU)	Inhibits glutathione reductase (GSR)	GSR	81
RSL3	Inhibits GPX4 in GSTZ1-deficient hepatoma cells	GPX4	142
Tiliroside	Inhibits TBK1-p62-Keap1-NRF2 pathway	GPX4, G6PD, FTH1	87
Metformin	Inhibits p62-Keap1-NRF2 pathway	HO-1	143
Camptothecin	Inhibits p62-Keap1-NRF2 pathway	GPX4, GSR, SLC7A11	144
Orlistat	Inhibits FASN/HIF1α/SLC7A11 pathway	SLC7A11	95
Withaferin A	Inhibits Keap1-NRF2 pathway	SLC7A11	145
Artesunate	Induces lysosome-mediated ferritinophagy	FTL	146
Caryophyllene oxide	Induces ferritinophagy by regulating the NCOA4/FTH1/LC3 pathway	NCOA4	147
Haloperidol	Enhances the expression of sigma receptor 1 (S1R)	S1R	148
Disulfiram (DSF)	Inhibits the compensatory NRF2 elevation	NRF2	149
Aspirin	Silences ACSL4 and induces GADD45B expression	ACSL4	150
Deferasirox (DFX)	Inhibits NF-κB activity	NF-κB	151

**Table 2:** Potential compounds or drugs for HCC therapy via triggering ferroptosis.

Compounds/ Drugs	Mechanism	Target	References
Gentian violet	Increases p53 levels	p53	152
Dihydroartemisinin (DHA)	Promotes PEBP1 protein expression	PEBP1	153
ZZW-115	Induces mitochondrial dysfunction with a ROS overproduction and downregulates key enzymes involved in the GPX-dependent antioxidant systems	TFAM	154
Anisomycin	Activates p38 MAPK through H3S10 phosphorylation and upregulates NCOA4	NCOA4	155
Electrophilic sesquiterpenes isolated from E. chinense L. (EChLESs)	Controls the expression of NCOA4 at the transcriptional and post-transcriptional levels	NCOA4	156
Schizocapsa plantaginea Hance I (SSPH I)	Elevates the expression of SLC7A5 and induces iron accumulation	SLC7A5, TFR and FPN	157
All-trans retinoic acid (ATRA)	Blocks GSH synthesis	GPX4, FTH1, GCLC, GCLM, SOD-1	158
PK11195	Inhibits TSPO-p62-Keap1-NRF2 pathway	GPX4, HO-1, NQO1, GCLC, GCLM	83
Polyphyllin I (PPI)	Inhibits NRF2/HO-1/GPX4 antioxidant axis	GPX4, HO-1	159
Scutellaria barbata	Promotes iron peroxidation and lipid ROS metabolism by regulating ferroptosis-related genes	GPX4, SLC7A11, IREB2, ACSL4	160
Solasonine	Destroys the glutathione redox system by suppression of GPX4 and glutathione synthetase (GSS)	GPX4, GSS	161
Parthenolide (PTL)	Increases GSH depletion, rapid oxidation of intracellular and mitochondrial thiols, mitochondrial dysfunction, and suppression of GPX4	GPX4	162
Heteronemin	Reduces the expression of GPX4	GPX4	163
Polyphyllin VI	Inhibits the STAT3/GPX4 axis	STAT3, GPX4	164
Atractylodin	Inhibits the expression of GPX4 and FTL and upregulates the expression of ACSL4 and TFR1	GPX4, FTL, ACSL4, TFR1	165
Aspirin	Triggers ferroptosis by restricting NF-κB-activated SLC7A11 transcription	SLC7A11	166
Solamargine	Inducing ferroptosis by downregulating MTCH1 expression	MTCH1	167

## Abbreviations

LIP: labile iron pool
TF: transferrin
TFR: transferrin receptor 1
STEAP3: STEAP family member 3
DMT1: divalent metal transporter 1
NCOA4: nuclear receptor coactivator 4
FPN: ferroportin
PUFA: polyunsaturated fatty acid
CoA: coenzyme A
PL: lysophosphatide
ACSL4: acyl-CoA synthetase long-chain family member 4
LPCAT3: lysophosphatidylcholine acyltransferase 3
PUFA-OOH: polyunsaturated fatty acid hydroperoxides
MUFA: monounsaturated fatty acid
ACSL3: acyl-CoA synthetase long-chain family member 3
GSH: glutathione
SLC7A11: solute carrier family 7 member 11
SLC3A2: solute carrier family 3 member 2
GPX4: glutathione peroxidase 4
Cys: cysteine
Gly: glycine
Glu: glutamic acid
Gln: glutamine
GSSG: glutathione disulfide
GSR: glutathione reductase
FSP1: ferroptosis suppressor protein 1
GCH-1: guanosine triphosphate cyclohydrolase 1
BH4: tetrahydrobiopterin
CoQ: coenzyme Q (also known as ubiquinone)
CoQH2: ubiquinol
DHODH: dihydroorotate dehydrogenase
GLS2: glutamine synthase 2
SLC38A1: solute carrier family 38 member 1
SLC1A5: solute carrier family 1 member 5
NEAT1: nuclear paraspeckle assembly transcript 1
MIOX: Myo-inositol oxygenase
HULC: highly upregulated in liver cancer
ATF4: activating transcription factor 4
ZNF8: Krüppel-associated box (KRAB)-type zinc-finger protein 8
lncRNA HEPFAL: hepatocellular carcinoma ferroptosis associative lncRNA
circIL4R: circ-interleukin-4 receptor
MELK: maternal-fetal leucine zipper kinase
YTHDF2: YTH N6-methyladenosine RNA-binding protein 2
lncFAL: ferroptosis-associated lncRNA
HDLBP: high-density lipoprotein-binding protein
SLC27A5: solute carrier family 27 member 5
TSPO: translocator protein
PCDH20: protocadherin 20
SIRT1: sirtuin 1
FNDC5: fibronectin type III domain containing 5
AdipoR1: adiponectin receptor 1
IGF2BP3: insulin-like growth factor 2 mRNA-binding protein 3
CPLX2: complexin II
CEP290: centrosomal protein 290
GSTZ1: glutathione S-transferase zeta 1
QSOX1: quiescin sulfhydryl oxidase 1
LCN2: lipocalin 2
CISD2: CDGSH iron sulfur domain 2
LIFR: leukemia inhibitory factor receptor
SHP-1: Src homology region 2 domain-containing phosphatase-1
STAT3: signal transducer and activator of transcription 3
MCL1: myeloid cell leukemia-1
BECN1: beclin 1
C8orf76: Chromosome 8 open reading frame 76
DAZAP1: deleted in azoospermia-associated protein 1
PNO1: RNA-binding protein partner of NOB1
S1R: Sigma-1 receptor
HIF-1α: hypoxia-inducible factor 1-alpha
FASN: fatty acid synthase
AKR1C3: Aldo-keto reductase 1C3
YAP: Yes-associated protein 1
TEAD1: TEA domain transcription factor 1
HBXIP: hepatitis B X-interacting protein
ETS1: ETS Proto‑Oncogene 1
SCD: stearoyl-CoA desaturase
SLC27A4: solute carrier family 27 member 4
MTCH1: mitochondrial carrier 1
